# Supplementary material for: An Intestinal Symbiotic Bacterial Strain of Oscheius chongmingensis Modulates Host Viability at Both Global and Post-Transcriptional Levels
Source: Int J Mol Sci. 2022 Nov 24;23(23):14692. doi: 10.3390/ijms232314692 (PMC9739912; doi:10.3390/ijms232314692)
Supplement: Supplementary file 1 [file ijms-23-14692-s001.zip › Supplemental Figures.pdf]

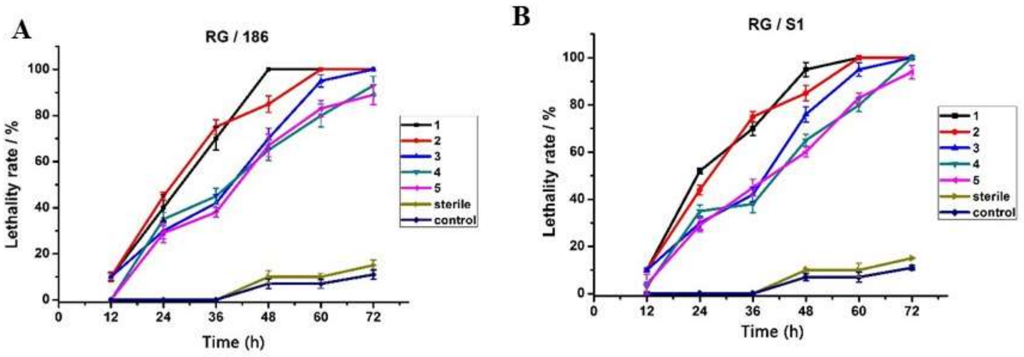

Figure S1. Pathogenicity changes of IJs of monoxenic nematode/bacterium combinations of O. rugaoensis associated respectively with S. nematodiphila S1(A) and S. nematodiphila DR186 (B) after multigenerational passage through G. mellonella in vivo.

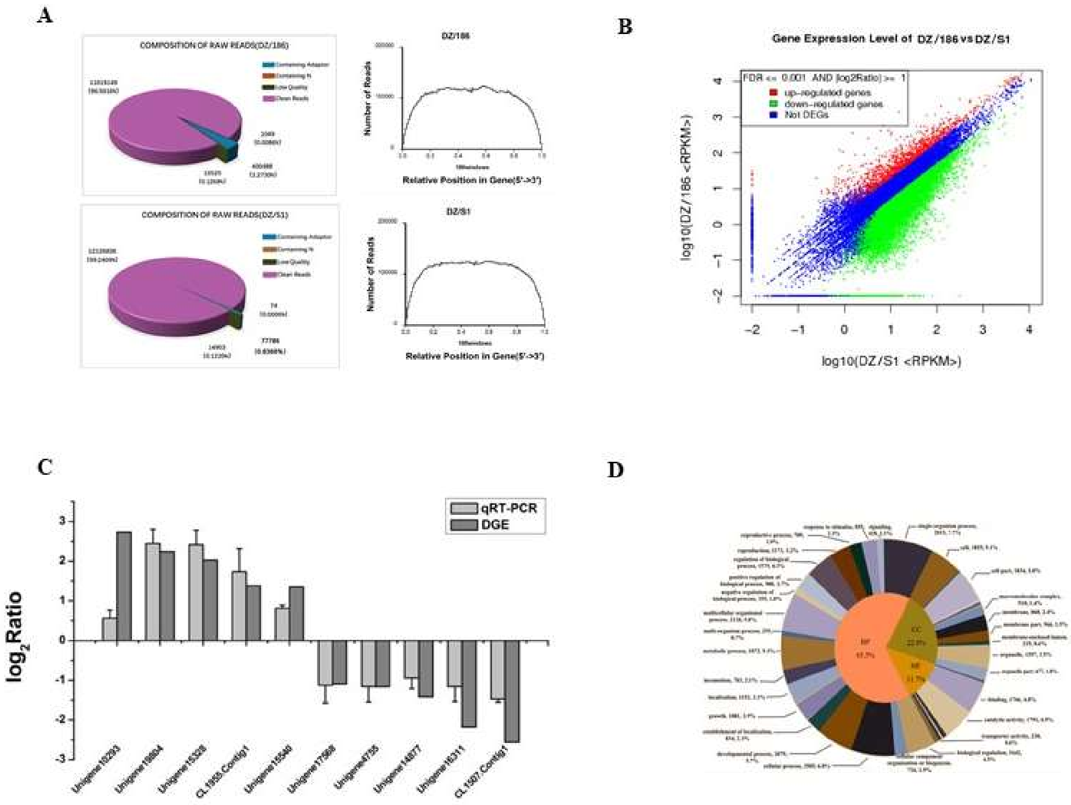

Figure S2. Global comparison analysis of mRNA sequencing data of native vs. non-native monoxenic O. chongmingensis; (A) Composition of total tags of the two DGE libraries of DZ/186 and DZ/S1. (B). DEG analysis between the two monoxenic combinations. (C). Validation of the RNA-Seq expression profiles of 10 genes randomly selected from among DEGs. (D). Gene ontology classification of all DEGs.

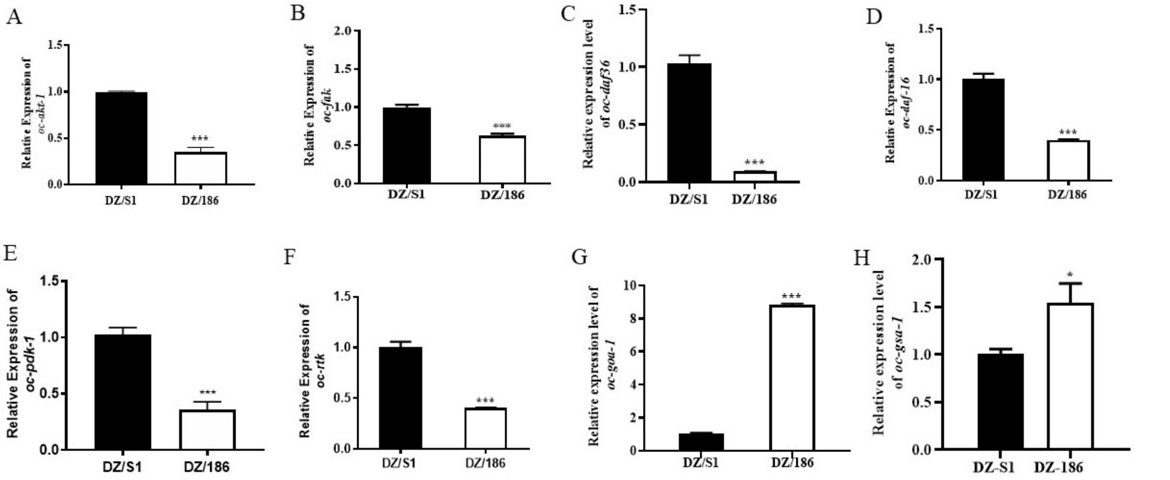

Figure S3. Expression levels of oc-akt-1 (A), oc-daf-16 (B), oc-daf-36(C), oc-fak (D), oc-pdk-1(E), and oc-rtk (F) are significantly downregulated in DZ/186 compared with DZ/S1, and those of oc-goa-1 (G) and oc-gsa-1 (H) are significantly upregulated in DZ/186.

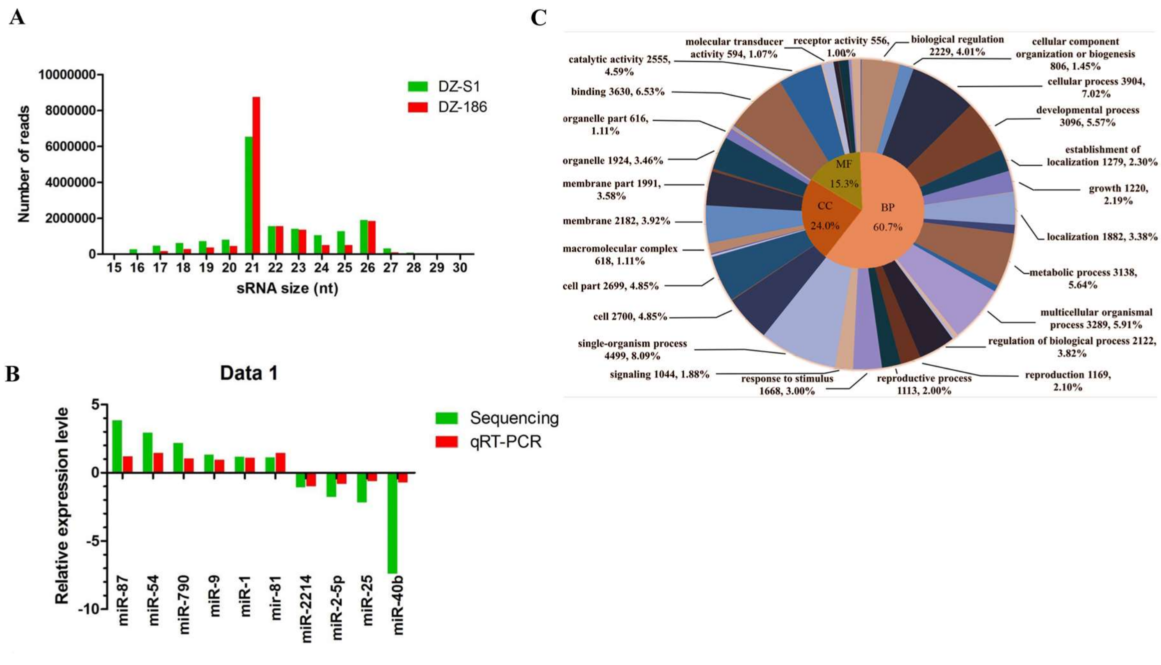

Figure S4. Global comparison analysis of miRNA sequencing data of native vs. non-native monoxenic O. chongmingensis; (A) Size distribution of small RNAs in DZ/S1 and DZ/186 libraries. (B) RT-qPCR validation of randomly-selected differentially expressed miRNAs. (C) Gene ontology classification of target transcripts for all differentially expressed miRNAs.
