# Supplementary material for: An Intestinal Symbiotic Bacterial Strain of Oscheius chongmingensis Modulates Host Viability at Both Global and Post-Transcriptional Levels
Source: Int J Mol Sci. 2022 Nov 24;23(23):14692. doi: 10.3390/ijms232314692 (PMC9739912; doi:10.3390/ijms232314692)
Supplement: Supplementary file 1 [file ijms-23-14692-s001.zip › Supplemental Tables.pdf]

Table S1. The survival of combinations of *Osheius chongmingensis* and *Serratia nematodiphila* treated with heat stress

Combination	No. of worms	No. of mortality	Median survival (hours)	Log-rank test
DZ/S1	90	48	24	*P=0.0039*
DZ/186	90	67	18	

***P* < 0.01 DZ/186 *vs* DZ/S1

Table S2. The survival of combinations of *O. chongmingensis* and *S. nematodiphila* treated with oxidative stress

Groups	No. of worms	No. of mortality	Median survival (hours)	Log-rank test
DZ/S1	90	88	15	*P*<0.0001
DZ/186	90	90	10.5	

***$P < 0.0001$ DZ/186 *vs* DZ/S1

Table S3. Summary of the differential expressed miRNAs in DZ/S1 and DZ/186

miRNA	Reads in DZ/S1	Reads in DZ-186	DZ/S1 -std	DZ/186 -std	Fold/change	p-value
lin-4	13708	26634	841.83	1691.68	1.01	0
miR-124	7862	40113	482.82	2547.82	2.40	0
miR-1	82032	178499	5037.74	11337.53	1.17	0
miR-2214	3518	1624	216.05	103.15	-1.07	8.99E-144
miR-228	24676	75759	1515.40	4811.90	1.67	0
miR-235	417	2825	25.61	179.43	2.81	0
miR-2-5p	14598	4147	896.49	263.40	-1.77	0
miR-34	2230	12681	136.95	805.45	2.56	0
miR-36a	734	1662	45.08	105.56	1.23	3.77E-89
miR-40b	7123	41	437.44	2.60	-7.39	0
miR-44	59484	151540	3653.03	9625.21	1.40	0
miR-48	3564	9211	218.87	585.05	1.42	0
miR-49	1731	6158	106.30	391.13	1.88	0
miR-50	4335	16203	266.22	1029.15	1.95	0
miR-54	4783	35345	293.73	2244.97	2.93	0
miR-56	1282	6224	78.73	395.32	2.33	0
miR-57	36035	116087	2212.98	7373.38	1.74	0
miR-61	4304	17330	264.32	1100.73	2.06	0
miR-63b	5450	11154	334.69	708.46	1.08	0
miR-71-5p	382341	677094	23480.29	43006.27	0.87	0
miR-72-5p	35678	120229	2191.05	7636.46	1.80	0
miR-787	493	1002	30.28	63.64	1.07	3.77E-44
miR-790	754	3302	46.30	209.73	2.18	0
miR-80	6628	16082	407.04	1021.46	1.33	0
miR-81-3p	146277	308316	8983.15	19582.98	1.12	0
miR-81-5p	527	1091	32.36	69.30	1.10	8.61E-50
miR-82	6959	16122	427.37	1024.00	1.26	0
miR-83	3127	10277	192.04	652.75	1.77	0
miR-84a	6817	17328	418.65	1100.60	1.39	0
miR-86	665	4594	40.84	291.79	2.84	0

Continued Table S3

miRNA	Reads in DZ/S1	Reads in DZ-186	DZ/S1 -std	DZ/186 -std	Fold/change	p-value
miR-87	186	2549	11.42	161.90	3.83	0
miR-255	8	54	0.4913	3.4299	2.80349	4.36E-10
n_miR-10	0	73	0.01	4.64	8.86	3.01E-23
n_miR-15	9	33	0.55	2.10	1.92	0.00011
n_miR-23	0	26	0.01	1.65	7.37	9.42E-09
n_miR-25	108	23	6.63	1.46	-2.18	0
n_miR-26	0	39	0.01	2.48	7.95	9.22E-13
n_miR-27	12	110	0.74	6.99	3.25	5.59E-22
n_miR-32	17	0	1.04	0.01	-6.71	1.03E-05

Table S4. Primers used for RT-qPCR analysis of expression level of unigenes or for RNA interference

unigene ID (Gene)	Primer sequence	
	Forward (5' to 3')	Reverse (5' to 3')
18S rDNA	CATCCAAGGAAGGCAGCAGG	CCGCAGCAATGACGATTTACAC
CL1507.Contig1	TTGTAGGCTCCCCTCTTTCTGC	CTTCGGTGGCAACACTCGTCT
CL1955.Contig1	TCCACAAGAACAGCACTGGTAGC	TTGAGGATGTCGGACGAGAAGA
Tnigene4755	GTTGTGTTCCGCAGGTCATCG	CTCCTTCTGTTCTTCGGGCTTGT
Tnigene10293	TCCGTTAGCTCCAGAACCTG	TTCATCGGAGGAAGTGGAGG
Tnigene14877	TTTCCGTTCTCTCCAGGTGG	CGTCCTCCTCAGTCTCCTTG
Tnigene15328	TCCTCAGCCTTTCGTCCATT	ACGAAGAGTGGGAACGAGTT
Tnigene15540	GAAGGACCTGCCTCATCACT	GCGAGACGAGCGTATTTGTA
Tnigene16311	CTCCCGTCGCTCAGGAAGAT	CCAATGGTGAAGAATGGGTTGA
Tnigene17568	GGAGGAGGAGTGATAGGCTC	ATACGATCAGGGAGTCAGCG
Tnigene19804	CGAATGAGCCAATGACTCCG	CCAAAGACCGTTCCAACCAG
6706 (*oc-akt-1*)	AAGGTTCTGGGCAAGGGTA	CGACGGTTTGGAAGCTGTA
oc-akt-1 RNAi	GCTGCAGCTGCAAGGAAAAGCGTT	CCTCGAGGCCACACATCATTTCGTA
18057 (*oc-daf-16*)	AGTTGCATCAATGAGCGTCG	TCTGAGCCTGGTTCTCTTCC
oc-daf-16-RNAi	CCTCGAGGCGGTGAATCGTCT	GCTGCAGCCGAATCTCCTTTGT
22051(*oc-daf-36*)	CACCCAGTTTGAAAGGGACATTT	ATTGACCTTGCTCTGTTTGTGAT
oc-daf-36 RNAi	CGAGCTCGGAGCTTGGGATTGTGGT	GGGGTACCCCTTTCAAACTGGGTG
10331 (*oc-fak*)	ATCCGAGTCCGTCTGGTTGC	AGCTCATAGGCCGATTGTGG
oc-fak -RNAi	AAGCTTAAGCACATAAGGCAT	CTGCAGGCAAGTGGACAATCA
15387 (*oc- goa-1*)	CTGGGAAGCAGATGGTAAGAGG	GCTGACAGTGGAGTGCAGGAAT
oc-goa-1-RNAi	GCTGCAGCCAAAACACAAACGATG	GGGTACCCTCAGGCAGACAAGACT
10127 (*oc- gsa-1*)	ATAGGTTACGGGAGTCTCTGGC	CTTCCTTGTCTTCGCTTTGGTC
oc-gsa-1-RNAi	AAGCTTAGAAAACACAAAGAGA	GGGTACCCGAGAGCGACTAAAAGC
12570 (*oc-gsa-1*)	GGAGTCGGGGAGGAAGGGA	TTGGCAAGAGGGGAGGTGT
oc-goa-1-RNAi	CCTCGAGGCTGTTTCCAGTGTT	GCTGCAGCCGTCTTGTCATTTT
CL395 (*oc-pdk-1*)	ATACGCCGCGCCGTGTCTACTAC	TTCCCCTCTTCACAGCCTCATCC
oc-pdk-1-RNAi	CCTCGAGCCTGTCCAGCCTAT	GCTGCAGCGTAGAACAAACGG
5468 (*oc-rtk*)	CTGTGTCGCTTTTTGTCCTG	AACCTTGTTTAGATTCGGGC
oc-rtk -RNAi	AAGCTTAGAAAACACAAAGAGA	CTGCAGAGAGCGATAGAACAGT

Table S5. Primers used for RT-qPCR analysis of expression of random selected and all the other miRNAs used in the present study.

miRNA	Primer sequence	
	Forward (5' to 3')	universal Reverse primer (5' to 3')
5S (reference)	GCTTTCCCTTCAGTTGTTCGG	GGCCAACCGCGAGAAGATG
miR-1	CGGACCTGGAATGTAAAGAAGTATGTA	GGCCAACCGCGAGAAGATG
miR-2-5p	ACGCACAAGCGGTGGATGATG	GGCCAACCGCGAGAAGATG
miR-40b	GCTCACCGGGTGTACTTCTTC	GGCCAACCGCGAGAAGATG
miR-54	GACCGTACCCGTAATCTTCATAG	GGCCAACCGCGAGAAGATG
miR-57	CCTACCCTGTAGTACCGAGCTGTGT	GGCCAACCGCGAGAAGATG
miR-71	GCTATCACTATTCTGTTTTTCGCAAA	GGCCAACCGCGAGAAGATG
miR-72-5p	GAGGCAAGATGTTGGCATAGCTG	GGCCAACCGCGAGAAGATG
miR-84a	GTCTGCGTGAGGTAGTTGAAAATGTTA	GGCCAACCGCGAGAAGATG
miR-87-3p	CACTCGTGAGCAAAGTTTCAGGT	GGCCAACCGCGAGAAGATG
miR-255	GACTAAACTGAAGAGATTTTTTACAG	GGCCAACCGCGAGAAGATG
miR-790-5p	GACTTGGCACTCGAGAACGAC	GGCCAACCGCGAGAAGATG
miR-2214	CACAACAGGTCCGGAGTGAATG	GGCCAACCGCGAGAAGATG

Table S6. Primers used for miRNA-target interaction identification in the present study.

Gene	Primer sequence	
	Forward (5' to 3')	Reverse (5' to 3')
oc-fak (cDNA)	ATCCGAGTCCGTCTGGTTGC	AGCTCATAGGCCGATTGTGG
oc-fak UTR	GATGTAGACAGTGCATTCCAGAAGTTTC	CGACGCGTCGGGAGAGGACACCGACCTTCT
oc-fak UTR-Mut	GATGTAGACAGTGGTAAGGTGAAGTTTC	CGACGCGTCGGGAGAGGACACCGACCTTCT
oc-goa-1（cDNA）	TTTTTTTTTGCAAAAACAA	CCAAGTTTGAGAGCTCGT
oc-goa-1 UTR	ATACAAACATATGATAAACTGTTTGCTTACAATG	CCGTTTTTACCTTTCTTCATTCGCATCGTTTGTG
oc-goa-1 UTR-Mut	ATACAAACATATGATAAACTGAAACGAATGAATGAAACAC	CCGTTTTTACCTTTCTTCATTCGCATCGTTTGTG
oc-gsa-1（cDNA）	AAGAGCACAATCGTCAAG	CAGACAACAACTTTATTT
oc-gsa-1 UTR	GCTATTGCTCATGCTCTACTTTCTTGCCG	GCAGTATGCCAAGGGCGTGCCG
oc-gsa-1 UTR-Mut	GCTATTGCTCATGCTCTACTTAGAACGGC	GCAGTATGCCAAGGGCGTGCCG
oc-pdk-1(cDNA)	CGCTGACTCGACTGCGTC	AATTATTATTATCATACA
oc-pdk-1 UTR	GGGTTTCTTCCTGGTGATTCCAGAATGCGTTAAATGCATTGGA	TTCTGGATAGTGTTGGAAGAAACCCAGAAGGCTAGAATACAAC
oc-pdk-1 UTR-Mut	GGGTTTCTTCCAACACTATCCAGAATGCGTTAAATGCATTGGA	TTCTGGATAGTGTTGGAAGAAACCCAGAAGGCTAGAATACAAC
oc-rtk (cDNA)	CTGTGTCGCTTTTTGTCCTG	AACCTTGTTTAGATTCGGGC
oc-rtk UTR	GACTAGTGGAACAACCGAATAACT	GAAGCTTAGAAGGCGTTTTACAGA
oc-rtk UTR-Mut	GACTAGTGGAACTTGGCTTTAACTGTTCTATCGCTCT	GAAGCTTAGAAGGCGTTTTACAGA
